# Primary care trainee nursing associates in England: a qualitative study of higher education institution perspectives

**DOI:** 10.1017/S146342362200072X

**Published:** 2023-01-09

**Authors:** Steve Robertson, Rachel King, Bethany Taylor, Sara Laker, Emily Wood, Michaela Senek, Angela Tod, Tony Ryan

**Affiliations:** 1 RCN Strategic Research Alliance, Division of Nursing & Midwifery, Health Sciences School, Barber House Annexe, 3a Clarkehouse Road, Sheffield S10 2LA, UK; 2 Leeds Beckett University, Leeds, UK; 3 Waterford Institute of Technology, Waterford, Ireland; 4 Department of Nursing, Winona State University, Winona, MN, USA

**Keywords:** education nursing, health workforce, staffing, United Kingdom, nursing associates, second level nursing

## Abstract

**Aim:**

To explore higher education institution (HEI) perspectives on the development and implementation of trainee nursing associates (NAs) in the primary care workforce in England.

**Background:**

Current shortages of primary health care staff have led to innovative skill mix approaches in attempts to maintain safe and effective care. In England, a new level of nursing practice, NAs, was introduced and joined the workforce in 2019. This role was envisaged as a way of bridging the skills gap between health care assistants and registered nurses and as an alternative route into registered nursing. However, there is limited evidence on programme development and implementation of trainee NAs within primary care settings and HEI perspectives on this.

**Methods:**

This paper draws from a larger qualitative study of HEI perspectives on the trainee NA programme. Twenty-seven staff involved in training NAs, from five HEIs across England, were interviewed from June to September 2021. The interview schedule specifically included questions relating to primary care. Data relating to primary care were extracted and analysed using a combined framework and thematic analysis approach.

**Findings:**

Three themes were developed: ‘Understanding the trainee role and requirements’, ‘Trainee support in primary care’ and ‘Skills and scope of practice’. It is apparent that a more limited understanding of the NA programme requirements can lead to difficulties in accessing the right support for trainees in primary care. This can create challenges for trainees in gaining the required competencies and uncertainty in understanding what constitutes a safe scope of practice within the role for both employers and trainees. It might be anticipated that as this new programme becomes more embedded in primary care, a greater understanding will develop, support will improve and the nature and scope of this new level of practice will become clearer.

## Introduction

The current shortage of medical and nursing staff within the United Kingdom (UK) health care workforce has been well documented (Buchan *et al.*, 2020; British Medical Association, 2021). This issue has been specifically highlighted in the UK primary care sector where the chronic shortage of general practitioners (GPs) has been associated with difficulties in accessing GP appointments (Royal College of General Practitioners, 2021). Similarly, concerns about the shortage of nurses within the UK community, nursing home and primary care sector have also been recognised and are predicted to become worse in the coming years partly due to an ageing workforce and the difficulties of recruiting internationally for posts that often require additional post-basic qualifications (Buchan *et al.*, 2020).

In England, one solution to help address the staffing shortage across the health and social care system was to introduce a new second-level nursing qualification – the nursing associate (NA). This level of practice is similar to that of ‘Licensed Practical Nurses’ in North America and ‘Enrolled Nurses’ in Australia and New Zealand. The suggestion for developing a new role was first recommended in the Shape of Caring review (Health Education England, 2015) which explored the future education and training needs of health care assistants (HCAs) and registered nurses (RNs). HCAs are non-registered members of the team who work alongside health professionals. In December 2015, the then Secretary of State for Health announced a plan to create a new nursing support role with the provisional title ‘nursing associate’ (Department of Health and Social Care, 2015) and in October 2016 Health Education England (HEE) was mandated to lead the piloting and development of the training for this role (Department of Health, 2016). Several drivers underpinned these decisions including the wish to facilitate career development for HCAs, the need to fill an identified clinical skills and knowledge gap between HCAs and RNs, and a desire to create an alternative route to becoming an RN.

The NA training is completed over two years at Foundation Degree level. The first two pilot groups of trainee NAs, based at 11 centres across England, commenced in 2017 and began joining the workforce in early 2019. While this pilot was funded directly by HEE, subsequent cohorts have mainly been funded through an apprenticeship model. As a consequence, trainees are primarily employees, many of whom are already been working as a HCA, who are expected to have time release for study leave (equivalent to one day a week) and for alternative placements (away from their employment base), rather than being students needing to gain experience in health and social care settings. In this sense, NA learning differs from that of traditional RNs in the UK and from second-level nurse educational status in North America and Australasia where learners are students not employees.

The requirement to undertake alternative placements, in a range of settings, links to a further important aspect of NA training. It was recognised from the outset that NAs should provide a flexible addition to the nursing workforce, able to work across any and all parts of the health and social care system. This would be achieved by ensuring trainees gain experiences across the four UK recognised fields of nursing (Adult, Child, Learning Disabilities and Mental Health) and that they develop experience of providing care ‘in hospital’, ‘close to home’ and ‘at home’ (Nursing and Midwifery Council, 2018). The ability of NAs to provide care in primary care contexts was therefore seen as crucial and linked to helping manage the increasing long-term conditions agenda, an important part of the National Health Service Long Term Plan (NHS, 2019).

Implicit in this requirement for NAs to be a flexible member of the nursing workforce is that trainees need to either be based with a primary care employer or be able to gain sufficient alternative experience within community and primary care settings. In England, community services within the NHS are provided through Community Trusts (these are sometimes part of a larger Trust that also includes hospital sector services) and consist of a range of services, such as district nursing, specialist long-term conditions care, preventative services like sexual health, child health services and others. Primary care on the other hand is delivered by general practices (GPs) that are independent, small- to medium-sized businesses contracted by the NHS to deliver services within a geographical or population area. Survey work commissioned by the National Institute for Health Research (NIHR) Policy Research Programme shows that Community Trusts tend to have smaller numbers of trainee NAs than Acute Trusts (Kessler *et al.*, 2021a). While this work provides no data on the employment of trainee NAs or qualified NAs in primary care (in GP), early nationally commissioned evaluations of the NA pilot programme noted that only 2% of trainees came from primary care (Traverse, 2019). However, following changes in the funding mechanisms for these trainees (ESFA, 2020), the numbers employed by the primary care sector are now increasing rapidly (Robertson *et al.*, 2022b).

An overview of the limited research that has been undertaken on the NA role in community and primary care settings (Robertson *et al.*, 2022b) highlights several issues of importance. First, it provides some evidence of the benefits that trainee and qualified NAs might bring to these sectors, such as trainees becoming more person-centred (less task focused), having increased skills in helping patients self-manage and freeing up RN time to engage in more complex care. Second, it notes that trainees in these sectors experience lower levels of support than their hospital-based peers. Third, it suggests that trainee NAs in community and primary care had a less clear sense of their professional identity, and of their future career direction, than their hospital-based peers. Finally, it flagged emerging concerns from the higher education institution (HEI) sector around the organisation and availability of placements, opportunities for peer support, and having adequate access to practice-based assessors (which is a requirement of the training in all clinical settings) for trainees employed in the primary care sector.

## Aim

This paper adds to the limited evidence on the programme development and implementation of trainee NAs within primary care settings. Specifically, it explores the perspectives from the HEI (university) sector on the development and implementation of trainee NAs within primary care.

## Method

This paper stems from a larger programme of research looking at the motivations, experiences and career aspirations for trainee NAs. Specifically, it is developed from a strand of this research programme that sought the perspectives of staff from HEIs. The design and methods of this HEI element of the research, and the specific aspects focusing on the primary care trainee NAs, are reported here using components from an abridged version of the Consolidated Criteria for Reporting Qualitative Research proposed by Tong *et al.* (2007).

### Study design

A descriptive and exploratory qualitative design within an interpretive framework was used to explore HEI perspectives on the development and implementation of the trainee NA programme. This paper focuses specifically on data relating to HEI perspectives on developing and implementing the programme in primary care settings.

### Participant selection

After ethics approval for the study was confirmed by the University of (blinded – Ref: 026 355), staff from five HEIs across England were recruited using a combined purposive and convenience sampling technique. Following initial contact with a senior academic in each of the five HEIs, invitations to participate were distributed to staff involved with the trainee NA programme. The research team then provided the study information sheet to those staff who expressed interest. This resulted in 27 staff agreeing to participate (Table 1) from two Universities in the North East, one in the North West, one in the Midlands and one in the South East. Written informed consent was obtained from all participants prior to data collection commencing. Participants were subsequently identified by a code to aid anonymity.

**Table 1.** Sample for England-wide HEI stakeholder interviews

Identifier	**Participants role** ^*^
**University 1**	
HEI1_S1	Senior management/leadership team TNA Programme
HEI1_S2	Senior management/leadership team TNA Programme
HEI1_S3	Senior management/leadership team Department of Nursing
HEI1_S4	Senior management/leadership team Department of Nursing
HEI1_S5	Senior management/leadership team TNA Programme
**University 2**	
HEI2_S1	Senior management/leadership team TNA Programme
HEI2_S2	Lecturer
HEI2_S3	Lecturer
HEI2_S4	Senior management/leadership team Department of Nursing
HEI2_S5	Senior management/leadership team TNA Programme
**University 3**	
HEI3_S1	Associate Professor
HEI3_S2	Associate Professor
HEI3_S3	Lecturer
HEI3_S4	Lecturer
HEI3_S5	Senior management/leadership team TNA Programme
**University 4**	
HEI4_S1	Senior management/leadership team TNA Programme
HEI4_S2	Senior Lecturer
HEI4_S3	Teaching Fellow
HEI4_S4	Senior management/leadership team Department of Nursing
HEI4_S5	Senior management/leadership team TNA Programme
HEI4_S6	Senior management/leadership team TNA Programme
**University 5**	
HEI5_S1	Senior management/leadership team TNA Programme
HEI5_S2	Senior management/leadership team Department of Nursing
HEI5_S3	Senior management/leadership team TNA Programme
HEI5_S4	Clinical Educator
HEI5_S5	Learning Development Facilitator
HEI5_S6	Senior management/leadership team TNA Programme

* To preserve anonymity, some roles have been given more generic titles as actual titles were very specific and may allow identification.

### Data collection

A semi-structured interview schedule for the wider HEI study was developed by drawing on existing NA literature and the team’s previous experience of research with trainee NAs (King et al., 2020, 2022; Robertson et al., 2021, 2022b). This schedule specifically included three questions, noted below, relating to trainee NAs from the private, independent and voluntary sector (which includes primary care) as previous research suggested there were particular concerns within this sector:

- What are the main motivations for commencing NA training in the Private, Independent and Voluntary Organisation sector?
- What are the main training concerns or challenges for trainee NAs from this sector?
- How has the HEI had to adapt to meet the needs of trainee NAs from this sector?

Due to the ongoing impact of COVID-19, interviews were conducted online by three members of the research team (two female and one male) between June and September 2021. Interviews lasted from 28 to 66 min (mean 48 min) and were audio-recorded and subsequently fully transcribed. The final part of the interview incorporated the three questions above. Data from these three questions, and other points in the interviews where primary care was specifically mentioned, form the basis for this paper. Malterud *et al.*’s (2016) concept of ‘information power’, rather than an attempt at data saturation, was used to make a judgement about the sufficiency of the sample and the data collected.

### Data analysis

Data analysis utilised a reflexive and iterative approach that combined aspects of framework analysis (Ritchie and Lewis, 2003) and thematic analysis (Braun and Clarke, 2006). All data relating to comments about the NA training programme in primary care were extracted into Quirkos^©^ software by one researcher [XX]. Through a process of iterative reading, key finding areas were initially identified by [XX], and these formed a frame that consisted of four parts: support; placements; understanding of the role and business planning; skills and scope. Data were then coded/categorised into each of these four elements using notes and signifying the interviews that linked to these codes/categories. An example of this early coding/categorising within one key area is provided in Table 2.

**Table 2.** Example of frame code/category development

Initial analysis into the frame	Second round analysis into the frame	Final analysis into theme and subthemes
Key area of frame: Understanding of the role and business planning		
Lack of understanding in primary care about what the role is and/or what is required [HEI1 S2, S3][HEI2 S3][HEI3 S3, S4, S5][HEI4 S2][HEI5 S2, S4]. HEI assisting with this understanding [HEI3 S5][HEI4 S5].	Less understanding of role and requirements in PC – [HEI1 S2, S3][HEI2 S3][HEI3 S3, S4, S5][HEI4 S2][HEI5 S2, S4]. HEI assisting with understanding [HEI3 S5][HEI4 S5]	Understanding the trainee role and requirements • Less understanding in Primary Care • Cost/benefit and incentives to develop NAs
Importance of cost and business model for GPs and HEE incentive [HEI3 S5][HEI4 S1, S4][HEI4 S6] ‘what’s in it for us’?][HEI5 S1]. Can be difficult for primary care to access the left over levy [HEI1 S1]	Cost-benefit and incentive [HEI3 S5][HEI4 S1, S4][HEI4 S6] ‘what’s in it for us’?][HEI5 S1]	
Some GPs are choosing to go down the assistant practitioner route rather than TNA so they don’t ‘lose somebody for two years’ [HEI1 S4].	Positive understanding – part of long-term planning [HEI2 S1][HEI4 S4] growing their own [HEI5 S4] staying with employer after [HEI2 S5] [HEI4 S5][HEI5 S4] reward for long service [HEI2 S5] freeing up PNs and improving patient care [HEI5 S1]	
Some GPs seeing sending an HCA onto the apprenticeship as a longer-term route to getting an RN when recruitment is difficult – long-term planning [HEI2 S1][HEI4 S4] growing their own [HEI5 S4] staying with employer after [HEI2 S5] [HEI4 S5][HEI5 S4] reward for long service [HEI2 S5].		

This initial frame and associated coding and categorisation were then considered by another team member [YY]. Following two rounds of iterative discussion, some re-categorisations were made; that is, some codes and categories were collapsed, or reassigned to different parts of the frame, and key areas refined and retitled as codes and categories were aligned to develop clear subthemes and themes to best present the data. Final subthemes and themes were agreed between XX and YY. These were sense-checked with the wider research team. Further critical interpretations of the data were then made during the development of this paper as findings were linked and integrated to previous research and policy, and authors discussed draft versions of the paper and specifically the interpretation of the findings in the discussion section.

## Findings

Three themes were developed from the analysis: understanding the trainee NA role and requirements, trainee support in primary care, and skills and scope of practice (Table 3).

**Table 3.** Theme and subtheme outline

Theme	Subthemes
Understanding the trainee NA role and requirements	• Less understanding in primary care • Cost/benefit and incentives to develop NAs
Trainee support in primary care	• From employer • System support/capacity
Skills and scope of practice	• Level of skill • Difficulty in obtaining skills • Role substitution/exploitation or blurring

### Understanding the trainee NA role and requirements

Participants generally agreed that there was limited understanding about NAs in primary care when compared to the hospital sector. In particular, participants mentioned the additional work involved in helping primary care employers understand what the requirements and expectations were when hosting a trainee NA. However, participants also recognised that fulfilling these requirements could be difficult for primary care employers, especially in the context of high service demand and limited staffing:

‘The main challenge is that they [primary care] don’t quite understand the role as much as the big Trusts do, and it takes a lot more work and building of partnerships to create that understanding’. [HEI2_S3]

‘There’s been teething problems in terms of making sure the ‘off-the-job’ elements are understood. Making sure trainees aren’t constantly in clinics all day. Realising they’re not your HCA anymore, they’re a trainee NA now. It’s realising that, to meet the requirements for this programme, they have to have that element of 20% ‘off-the-job’ learning, they need supervised practice, they need supernumerary time. I think that’s been challenging for the GP surgeries’. [HEI3_S3]

Implicit in these accounts were concerns that the requirements, especially the need to have time release and for trainees to have access to a suitably qualified practice assessor in the workplace (a requirement for trainees in all clinical settings), were often not fully understood in advance by primary care employers, creating challenges for both the HEI and the employer:

‘There’s been increased interest from primary care. But there are huge challenges there because they don’t always fully appreciate that they’re going to lose their trainee when they complete their alternative placement hours. We’ve seen quite a lot of people being put forward and then an awful lot of backtracking when the penny drops. Which is a real shame’. [HEI1_S4]

‘One difficulty we’ve had with primary care is that whilst they want to send somebody on this [NA] programme, they don’t necessarily realise the things they have to have in place to support that trainee. And it’s not until we’re contacting them that you’re finding they’ve not got a practice assessor, they’ve not got a lot of things in place and we then have to train-up to get those things in place’. [HEI5_S2]

However, there was also recognition that some primary care employers were understanding and acknowledging the potential benefits NAs could bring and planning accordingly. This was often linked by participants to employers being able to take a longer term view, particularly in relation to how NAs might help with medium or longer-term staffing concerns within primary care:

‘Some of the smaller employers have said, “We just can’t get qualified staff, registered nurses, to come and work in the practice”. So they’re wanting to train up the HCAs they’ve got to fill those posts. Long-term planning really. So two or three years down the line, “We’ve got a qualified nurse”’. [HEI2_S1]

‘Especially in GP surgeries, where this may be someone who’s worked with them for many years as a HCA or whatever. They see them blossoming and developing and I think they feel they can really shape them into fulfilling the role that they want their NA to be’. [HEI5_S4]

Several participants felt strongly that it was therefore part of their role to promote the benefits that training NAs might bring to primary care employers. It seemed especially important to be able to help primary care employers see the link between promoting career development for their staff and ensuring their workforce for the future in a way that limited the cost to the practice and improved service delivery:

‘I think what’s really important is sell the role, that actually you’re investing in your staff and you’re growing your own staff and you’re equipping them with better skills for them to come back to the surgery with and implement within the workplace’. [HEI4_S1]

‘It’s more palatable to say to a GP, “We’re going to take your HCA and we’re going to transform them into a nurse. It’s going to be over two years and it can be an apprenticeship and we’ll sort out the levy, so you don’t have to pay. You’ll still get some of their time.” Rather than saying, “We’re going to take them away for three years and you’re hardly going to see them at all.” It’s a business thing’. [HEI4_S4]

Although promoting the NA role to GP employers was clearly seen as important, some quotes here might suggest that NAs would provide an alternative, or even an equivalent role, to RNs within the primary care context. While NAs may take on some tasks previously undertaken by RN-qualified practice nurses, the role is not intended to be one of substitution. As such, enthusiastic HEI promotion of the role should take care not to give the impression that releasing staff to complete NA training will provide the equivalent of an RN. Rather, it should focus on how NAs would fit within modern primary care skill mix and emphasise role boundaries. These challenges around the scope of the NA role are considered further in a later theme.

A fuller understanding of the NA role and purpose, and the training commitments and requirements, leads to a necessary consideration of what the support needs might be for trainees within primary care.

### Trainee support in primary care

Participants highlighted that the support provided to trainee NAs by primary care employers varied. As mentioned earlier, this mainly related to the support provided by practices to ensure the required opportunities for protected learning time or alternative placement release were met. Again though, the specific reasons for struggling to meet these support challenges in the primary care context were noted by some participants:

‘The GP surgeries are really inconsistent. It’s the first time that we’ve had primary care trainees with us and there’s two practices that are brilliant and their students are supernumerary the entire time of the course. Whereas the other two practices, they’re booking their trainees into doing clinics three full days a week so they’ve no leeway to go and spend a day with a district nurse or anything like that because they’re not being flexible with their clinics. We’ve had a lot of conversations with them and it’s just down to the practice managers saying that they can’t release them’. [HEI3_S4]

‘If it’s a GP surgery, the GP and the practice manager don’t want to send the member of staff [trainee NA] off. I think that’s because in small practices they just don’t have the staff around that are easily replaced. In a hospital, it’s quite easy to send trainee NA off when you’ve got eight other HCAs on the same ward’. [HEI4_S1]

As well as difficulties in obtaining time release, the nature of primary care workplaces, particularly the size of the team, also limited the supervisory and peer support available from nursing colleagues. This could leave NA trainees in primary care somewhat isolated and unsupported:

‘I think what we tend to find with our independent organisations, like primary care, is that they maybe don’t have that many registered nurses that can support the trainees’. [HEI3_S3]

‘They [large Trusts] already work with a lot of student nurses as well. Whereas trainees are quite isolated in the GP practices’. [HEI3_S4]

Beyond the support offered, or not, by employers, there were wider issues noted by participants relating to how the system itself can restrict available support for trainees within primary care contexts. The size and existing educational structures in larger hospital Trusts were seen to provide a support framework for trainees that was not readily available to primary care trainees; although the development of Primary Care Networks (PCNs, groups of GP practices working collaboratively) could help ameliorate this issue:

‘We’ve had a lot more GP practices in the last few cohorts. That brings its own challenges, because they don’t have the infrastructure wrapped around them that big [hospital] organisations have. Normally, we would just assume that mandatory training and everything like that would be done for [hospital Trust] because they’ve got a training department. But that’s not the case for GP practices’. [HEI5_S4]

‘I think they’ve [primary care] just not got the safety net. They don’t have the clinical educators who work side-by-side with you if you’ve got particular problems. They don’t have the capacity to impact as much as the big Trusts that have a separate clinical educator who can come in and make an action plan if something goes wrong, who is more linked to the university and more linked to the education side of things and knows what they’re looking out for’. [HEI2_S3]

‘What’s good is that within primary care, because there’s the Primary Care Networks, they [trainees] can be placed at different surgeries. It might not necessarily be the surgery the trainee comes from. The nurse education lead can liaise with other surgeries within that PCN and place trainees within that PCN rather than just one surgery’. [HEI4_S1]

Most participants noted their HEI had established support meetings, some required by the training body, such as the tripartite meetings between employer, HEI and trainee, and some additional to help organise placements or address queries that might have arisen. Again, these were familiar educational structures in larger Trusts and hospital contexts but less so in primary care which could lead to reduced involvement:

‘We have these drop-in meetings every month where if any employers or mentors or assessors have got any questions about the [NA] course, they can just drop in and ask us, but the GPs never attend any of them. But they’re the ones where we’re having the issues with!’ [HEI3_S4]

Adequate understanding and appropriate support is important for several reasons one of which links to trainees being able to gain the required skills and knowledge competencies to operate safely within a well-defined scope of practice.

### Skills and scope of practice

Some participants noted that primary care trainees had a limited basic nursing tasks skill set relative to hospital trainees. Conversely, there was recognition that some already have advanced competencies:

‘I tend to forget that some might not have really basic nursing skills. For instance, coming from a GP practice, you don’t routinely bed bath someone. […] I have to keep reminding myself, it’s about all those proficiencies and skills. If you work in a GP practice, you’re not really doing it’. [HEI1_S2]

‘In the GP surgery they [trainees] have phlebotomy skills, are able to run diabetes clinics and those sorts of clinics. And they can already do things like give injections, take ECGs, take bloods’. [HEI2_S3]

A more pressing issue than the level of skills that trainees from primary care commenced with was the restricted opportunities available to develop the required skills and knowledge competencies. This was related to both the breadth of experience that could be gained within a small primary care employer and having to gain many competencies in a short period of time during alternative, hospital-based, placements. In addition, learning skills that would likely never be used in primary care also seemed to be a concern:

‘When we’ve got trainees working in primary care they’re not going to be exposed to the opportunities that somebody in an acute setting would be […] Trainees working in primary care, in GP surgeries, they rely on that three-week block [alternative placement] to try and get everything done’. [HEI4_S3]

‘Some skills they have to learn, trainees in a GP practice will never, ever have to do that. Like there’s a section on caring for dying patients and last rites; well, a NA in a GP practice, would never see that in their day-to-day work […] So, there were certain things, which they’ll never, ever use, which makes it a bit crazy that we’re training them to do that’. [HEI2_S1]

Finally, there were interesting points raised about the possibility of role substitution for trainees in primary care once they have qualified. This was seen not only to be about taking on roles previously undertaken by RNs but also related to accountability in primary care where, as an independent business, lines of accountability could be more ambiguous:

‘For example, in GP practices, they say “I did this course, the smear test course” or “we did this course.” It’s absolutely great that they’re moving onwards and upwards but my fear is that they’re just becoming RNs […] I think there’s an issue with the expansion of the HCA role within those smaller areas. I suppose they have more power or more ability to increase those roles because the accountability is different where the GPs or the practice holder take accountability for what members of staff do’. [HEI2_S3]

‘I think the social care sector want a cheap alternative to nurses. I think GPs are the same. They want someone who can do a clinic, be accountable for it, but not have to be necessarily overseen by a more expensive RN’. [HEI3_S5]

The pre-existing skill set of trainees can vary widely between those based in primary care and those from hospital settings. Furthermore, opportunities to gain certain required proficiencies during training were limited in primary care settings. Concerns around the scope of this new level of practice, boundary blurring and accountability were also noted.

## Discussion

Early work on the introduction of the trainee NA programme highlighted the difficulties that a limited understanding of the trainee requirements, and the role itself, created for employers (Vanson and Bidey, 2019; Kessler *et al.*, 2020) and for trainees (Coghill, 2018; Vanson and Bidey, 2019; King *et al.*, 2020). More recent work suggests that this understanding has improved, that some of the early problems are beginning to resolve, and that many employers plan to continue embedding the NA role (Kessler *et al.*, 2021a; Robertson *et al.*, 2022a). However, findings here suggest that concerns remain about the level of understanding regarding requirements and expectations for trainee NAs among many primary care employers. The most likely reason for this is that uptake of the NA programme has taken place later in primary care than in hospital and combined hospital and Community trust contexts. The complexity of introducing new roles into primary care, and the need for better recognition of the factors affecting the assimilation of these roles, has been previously noted (Nelson *et al.*, 2019). Yet, there are precedents for managing such skill mix assimilation. The development of advanced nurse practitioners initially created challenges, concerns and even resistance, but they are now an accepted, understood and mainly appreciated part of the primary care workforce (Greenwood, 2019). Similarly, the introduction and assimilation of Physician Associates in primary care has proved to be a complex but clinically and cost-effective approach to skill mix in the UK primary care context (Drennan *et al.*, 2015). Findings here demonstrate that HEIs can help promote understanding of the requirements and advantages that these new trainee NAs might bring to the primary care team. However, care should be taken to ensure that any promotion of the role does not give the impression that releasing staff for NA training will provide the GP practice with the equivalent of a RN-qualified practice nurse on training completion. Future research might look in more detail at the specific factors influencing the understanding and assimilation of trainee and qualified NAs in primary care.

While supporting trainee NAs may begin by promoting an understanding of the requirements and scope of role, this alone is not enough. Kessler *et al.* (2021b) note that the ability of trainee NAs to acquire required competencies is dependent on their status within clinical teams and on workforce pressures. Evidence here suggests that the limited size, and independent business nature, of many primary care workplaces and teams can mean that opportunities for appropriate supervision and adequate protected learning time for trainees are constrained. Trainee NAs are in a different situation to student nurses in that they are primarily employees, and this has implications in the practice setting, particularly when service demand is high and clinical need takes precedence over learning experiences (Kessler *et al.*, 2021b; Robertson *et al.*, 2022a). Trainee NAs in hospital Trust settings work in larger teams and often have access to wider systems of organisational support, such as clinical educators, whom they can turn to if problems arise. These systems serve as a bridge between the clinical setting and the HEI providing front-line support for trainee NAs (Robertson *et al.*, 2022a). Within primary care, such wider systems of nursing educational support are not always well established. However, although not a directly intended objective, the establishment of collaborative PCNs in England (Fisher *et al.*, 2019) could provide an excellent framework for expanding this wider system support for trainee NAs as they link primary care with other community health and nursing services. Further work exploring current educational support practices among PCNs in order to disseminate and extend best practice would be helpful.

While difficulties in gaining required competencies is generally recognised among trainee NAs (Kessler *et al.*, 2021b), findings here suggest this can be compounded in primary care as assessors might be less available and certain competencies can only be gained in hospital settings where placement time is limited. The value of attaining competencies that do not relate to the primary care context was also noted. This raises questions about how the purpose of NA training is perceived by different stakeholders. While envisaged in policy terms as being about the production of a generic worker within the health care system, primary care employers might be more interested in the development of a trainee specifically skilled to perform effectively in the GP setting. This is especially the case if GPs are developing HCAs as part of a long-term staffing plan in the context of nursing shortages in primary care as some were noted to be doing. HEIs were often left managing the tensions that occurred when a shared sense of purpose in relation to NA training was absent, and future work could be undertaken to consider how best to develop a shared sense of purpose between policy implementers and primary care employers.

As a new role, there was uncertainty noted about the scope of this level of practice. In blurring the boundaries between HCAs and RNs, it was recognised that some trainees were undertaking similar roles to RNs in primary care, with potential implications for ensuring lines of accountability and adherence to regulatory standards. Previous work has identified that GP managers recognise the importance of new roles in primary care, and that this is strongly motivated by a desire to increase appointment availability, release GP time and therefore increase business efficiency (Gibson *et al.*, 2020). However, this requires those undertaking new roles to work more autonomously. Nelson *et al.* (2019) suggest the risks around this increased autonomy can be daunting for those in new primary care roles, particularly where there is ambiguity over role definition and purpose and a level of role substitution. It was apparent that there were concerns from HEI participants that the level of autonomous and independent working for trainee (and subsequently qualified) NAs in primary care was not always clear. Future research could help establish what constitutes the current scope of practice boundaries, what are the expected and actual levels of independent working, and where and how clear the lines of responsibility are for both trainee and qualified NAs in primary care. This research should incorporate the perspective of primary care trainee NAs themselves, a voice that is currently largely absent in the literature.

### Limitations

There are limitations to the current study. Data collection was limited to five HEIs so may not reflect all the issues occurring when developing and implementing the NA programme in primary care. In addition, the primary care focus only formed one aspect of the larger study with limited emphasis placed on this in the interview topic guide. Nevertheless, the multisite nature of the study, and having some specific emphasis on HEI perspectives of implementing the NA programme in primary care, moves beyond most previous research that has focused generically on trainee NAs or on single-site HEI studies.

## Conclusion

It is apparent that the limited understanding of NA programme requirements can lead to difficulties in getting or accessing the right support for NA trainees in primary care. In turn, this can create challenges in gaining required competencies and uncertainty in understanding what constitutes a safe scope of practice within the role for both employers and trainees.

It can be anticipated that as the NA programme becomes more embedded in primary care that greater understanding will develop, support will improve and the nature and scope of this new level of practice will become clearer. However, such progress does not happen by chance. While HEI participants were mainly passionate about promoting an understanding of the NA programme and training requirements, putting this understanding into action requires wider change. Recognition of the staffing and the service and business demand needs in primary care is required if a better balance is to be made between immediate service provision expectations and longer-term learning requirements for trainee NAs. In addition, system-level support, perhaps through the PCNs, could ensure the effective use of resources to help trainees access appropriate and timely clinical support and the learning opportunities to develop required competencies. Finally, as the number of trainees (and therefore future NAs) in primary care is increasing, it would seem timely to attempt to bring clarity to the scope of this level of practice in primary care in order to ensure that their place within the team is well understood, that they are not operating as a form of cheap substitution, and that their practice is safe and effective.
